# Role self-ascription of professionals conducting advance care planning conversations: A thematic analysis

**DOI:** 10.1177/02692163251331168

**Published:** 2025-04-25

**Authors:** Anca-Cristina Sterie, Mathieu Bernard, Ralf J Jox, Eve Rubli Truchard

**Affiliations:** 1 Chair of Geriatric Palliative Care, Palliative and Supportive Care Service and Service of Geriatric Medicine and Geriatric Rehabilitation, Lausanne University Hospital and University of Lausanne, Lausanne, Switzerland; 2 Chair of Palliative Psychology, Service of Palliative and Supportive Care, Lausanne University Hospital and University of Lausanne, Lausanne, Switzerland; 3 Service of Palliative and Supportive Care, Lausanne University Hospital and University of Lausanne, Lausanne, Switzerland; 4 Institute of Humanities in Medicine, Lausanne University Hospital and University of Lausanne, Lausanne, Switzerland; 5 Service of Geriatric Medicine and Geriatric Rehabilitation, Lausanne University Hospital and University of Lausanne, Lausanne, Switzerland

**Keywords:** Advance care planning, communication, role, palliative care

## Abstract

**Background:**

During advance care planning, individuals can benefit from the support of a healthcare professional to navigate the intricacies of decision-making. There are specific roles to be played at each level of the process. Evidence is lacking about how professionals understand their role when conducting advance care planning conversations.

**Aim:**

To explore how professionals perceive, define and describe their role when conducting advance care planning conversations.

**Design:**

We conducted this exploratory cross-sectional study in Switzerland from November 2019 to June 2020 by using semi-structured interviews, which were transcribed and thematically analysed with an inductive approach.

**Participants:**

Fourteen professionals having received a training on advance care planning in Switzerland.

**Results:**

We identified three themes: (1) role typology; (2) individual-centred and (3) professional-centred aspects related to role ascription. Roles that professionals undertake were aggregated in two overarching categories, ‘facilitators’ and ‘counsellors’, according to whether they prioritise individual’s capacity to decide for themselves or their need to receive guidance towards a particular decision. In practice, roles fluctuate between these categories, according to the individuals (to what extent they are informed and eager to engage in autonomous decisions, their communication capacity and desires) or the professional (main profession and involvement in the person’s care plan).

**Conclusions:**

Advance care planning requires professionals to be very adaptable and flexible in order to identify the role that they can play in each situation. Training needs to take into consideration this complexity and address it explicitly.

**What is already known about the topic?**

- One of the specificities of advance care planning resides in the fact that individuals receive the support of a third party to help them navigate the intricacies of decision-making
- The role of leading advance care planning conversations is a critical element of the process and requiring particular competences
- Many professionals identify the need to clarify their own role within advance care planning and associate this to a barrier in practicing and implementing advance care planning

**What this paper adds?**

- Findings show how professionals who conduct advance care planning conversations perceive, define and describe their role during advance care planning (what can be identified as ‘role self-ascription’)
- Role shifts dynamically between two categories, ‘facilitators’ and ‘counsellors’, showing that role borders are permeable and that professionals juggle with a dual attitude
- Certain professions were considered more competent to conduct advance care planning, such as being a clinician or a medical specialist

**Implications for practice, theory or policy**

- Advance care planning training needs to take into consideration that professionals are required to be very adaptable and flexible throughout the process in order to identify the role that they can invest in each situation

## Background

Advance care planning enables individuals to make health-related decisions in anticipation of a situation in which they would be incapable of doing so spontaneously.^1^ During advance care planning, people receive information about potential health trajectories and treatment options, identify values that are relevant for defining goals of care and what medical outcomes might or might not be acceptable, and often record their results and decisions for future care.^1^ Evidence compiled by systematic reviews highlights several positive effects of advance care planning, particularly in the context of terminal illness, such as reducing hospitalisation, increasing use of palliative services, increasing concordance between actual care and people’s care wishes, and decreasing use of life-sustaining treatment that would drastically lower quality of life.^2,3^ Other reviews suggest shortcomings in the practice.^4^ Studies show that outcomes of advance care planning are influenced by legislations, institutional policies and cultural factors^5^ as well by numerous barriers, such as lack of training and time to conduct conversations,^6^ its inadequate implementation (e.g. offering it too late).^7^

While advance care planning programmes exist in which patients are only provided with decision aids and complete the process by themselves, in most cases, advance care planning relies on a healthcare professional who supports patients to understand, explore and make decisions.^5^ Recognising the complexity and life-and-death implications of the decisions made during this process, healthcare professionals need to be trained to be able to conduct advance care planning conversations.^1^ Internationally, these trainings can be designed only narrowly for professionals with clinical duties, mainly doctors^8^ and nurses,^9^ as well as interdisciplinary, open to any professionals working in the healthcare domain, such as social workers.^10^ Certain programmes invest only in non-healthcare or clinical professionals, called ‘patient navigators’.^11^ No guidance is given as to the background of professionals, except to the fact that they should be ‘trained’.^1^

There are specific roles to be played at each moment of advance care planning, that is, when initiating or conducting it, as well as when documenting preferences. The European Association for Palliative Care^1^ identified six ‘recommended roles and tasks’ that reflect this complexity: adopting a person-centred approach; having the necessary skills and being open to talk about diagnosis, prognosis, death and dying; providing individuals with clear and coherent information; possibility for non-physician facilitators to be involved in advance care planning; possibility of it being initiated in and outside of health-care settings; involving health-care providers to discuss diagnosis, prognosis, medical options. Specific studies have also looked more in depth into the issue of advance care planning professional roles. Most often, they conjugate roles and responsibilities according to the main profession of the person conducting advance care planning: nurses,^12^ physicians,^13^ allied health professionals,^14^ care assistants,^15^ social workers,^16^ chaplains.^17^

Throughout the literature, the role of leading advance care planning conversations is identified as a critical element of the process and requiring particular competences. As Carr et al.^18^ showed, self-efficacy is statistically associated with an increased number of conducted conversations, while knowledge isn’t; this shows that practices and engagement are modelled by what facilitators think about themselves. Despite existing guidance, several studies point out the fact that many professionals identify the need to clarify their own role within advance care planning and associate this to a barrier in practicing and implementing it.^6,19,20^ This highlights the evermore relevance of looking into how professionals understand their roles and how these conceptions are being constructed (e.g. during training but also with experience),

### Aim

Our aim was to explore how professionals who conduct advance care planning conversations perceive, define and describe their role during advance care planning (what can be identified as ‘role self-ascription’). Our focus has been exclusively on the act of conducting advance care planning, and not connected to other activities such as teaching or awareness raising.

## Methods

### Study design

We conducted an exploratory cross-sectional study^21^ in Switzerland from November 2019 to June 2020 using semi-structured interviews.

### Setting

In Switzerland, the legal adoption of binding ‘advance directives’ in 2013 created an impetus for the development of advance care planning^22^ and several regional initiatives for implementation and training of professionals, especially in Zurich,^23^ Lausanne,^24,25^ and Geneva.^26^ The training is offered to ‘health and social work professionals, holding a university degree, or physicians with experience in communication with patients’.^27^ Concretely, participants have come from the following backgrounds: nurses, physicians, social workers, medical secretaries, health sociologists, psychologists, chaplains, death doulas.

### Participant population

Inclusion criteria consisted of (i) having received a formal training in Switzerland in the past 10 years for conducting advance care planning, (ii) having conducted at least one advance care planning conversation in Switzerland, (iii) in a Swiss national language (French, German, Italian).

### Participant sampling

A purposeful sampling strategy was used. Participants were identified initially by online research of registered professionals conducting advance care planning in Switzerland, who were all (*n* = 13) contacted by email. Given the low number of those who accepted to participate (*n* = 10), more were identified among those who did training in the Lausanne area and having done at least one advance care planning conversation since (*n* = 8).

Participants received written and verbal information via e-mail; those interested to participate were asked to sign an informed consent form.

### Data collection

We employed semi-structured interviews as a resource to access participants’ own understandings and formulations about roles. Interviews were conducted in French and German by native speakers, took place via telephone and virtually (due to COVID-19 restrictions), and were audio-recorded. Interviews in French were transcribed verbatim and those in German were transcribed directly in French. The interview guide was developed by ACS (see Supplemental Material 1). It gathered basic sociodemographic information and explored the participant’s advance care planning training and current practice, their perceptions about advance care planning and their role within it, and practices, challenges and resources related to communicating with beneficiaries during advance care planning. Participants were invited to open share their experiences and perceptions, without referring to hypothesis or expected results. It was initially tested on two individuals (not included in the sample), after which minor modifications were done.

### Data analysis

Interview data was analysed with reflexive thematic analysis.^28,29^ Initially, ACS (a health sociologist) read the transcripts repeatedly to develop a broad sense of perceptions that facilitators have about their role. Two conversations were inductively coded by the three authors (ACS, MB, a palliative care psychologist and researcher and ERT a medical doctor specialised in geriatrics), independently and in parallel. Codes were assigned to sentences or paragraphs containing several sentences. The team compared and reviewed the codes. This stage was extremely useful in identifying aspects from the interview that relate to how facilitators perceive and experience their roles. We found that establishing a certain level of coherence at this stage was essential, particularly given our different disciplines. Then, ACS conducted an initial inductive descriptive coding of the whole data. ACS subsequently identified and grouped similar codes into broad themes, which she then then differentiated into sub-themes when relevant (i.e. when the theme contained too diverse aspects). Themes were not mutually exclusive of each other and could occur in tandem. Driven by the way in which participants talked about the topic, the themes revolved around two dimensions: (i) what are the roles of facilitators and how they are expressed or made manifest (Table 1), and (ii) the aspects that led to or characterised these roles (Tables 2 and 3). As such, the themes were ‘conceptualised as patterns of shared meaning, organised a central concept’.^29^

**Table 1.** Characteristics of study participants.

Characteristic	Number (*N* = 14)
Professional background	
Non-clinical	1
Ethicist	
Clinical	
Nurse	10
Physician	2
Psychotherapist	1
Jurisdictions in which the training was done	
Vaud	5
Zurich	12
Language in which ACP is conducted	
French	10
German	7
English	1
Number of years since ACP training	
Less than 2 years	4
More than 2 years	10
Estimated number of ACPs done since training	
1–5	3
Up to 10	0
More than 10	11
Setting in which ACPs are/have been conducted	
Palliative care	10
Intensive care	2
Oncology	1
At home	4

**Table 2.** Role typology.

Theme: role category	Sub-theme: role category sub-type (approach)	Selected quotes
The facilitator	Facilitating	If we start from the idea that a person knows well enough what is good for herself, I see myself as a facilitator (. . .) In my role as facilitator, I am not responsible of what happens after. (F1) Being there as a facilitator, to start the discussion. And then I withdraw from the discussion, I let people talk and I find that wonderful (F3)
	Accompanying	We use the term ‘facilitator’ because we accompany but we don’t provide counsel (F2) It’s not just a consultancy work, it’s a philosophy. A holistic history about how I understand care. How I understand companionship in the life process. I don’t think of myself as omniscient. I accompany people on the road they want to take and I use my expertise to help (F8).
	Helping	I start from the idea that people know very well what they want, and I help them articulate this orally and in writing (F1) We adapt to the patient’s needs. Maybe we help formulate but we won’t make him do something he doesn’t want (F5)
	Translating	[I see myself as] a translator as well. Because we write the document. And the patient say wow it’s crazy, it’s exactly how I would have written it (F4) I had to translate a lot of the ACP form so that the patient understands (F14)
The counsellor	Coaching	I am in the role of the coach. I must help the other person to find the right solution. (F9) I see myself as a coach. (. . .) My task is the following: if something is contradictory or technically incorrect, then I should intervene in a corrective way and signal it (F10)
	Counselling	I can’t force the person to make a decision with which I agree. But I can make remarks and tell them what I think, as a counsellor. For example, if a person wants palliative measures but has good chances of a positive outcome, I can make a remark (F9)
	Guiding	There are people who are very lost and in those cases one has to be more of a guide, more clear about what is possible (F4) I guide people in their process. I ask critical questions (. . .) I drive the car and you sit in it and take what you need (F12)
Shifting roles		There are people who are very lost and in those cases one has to be more of a guide, more clear about what is possible. (. . .) There are people who are very determined. In those cases, one just has to listen (F4) There are people whose ideas are sufficiently clear. I try to withdraw as much as possible and just make them realise when there are incoherences (. . .) Others clearly need examples. So in that case, I am more of a guide (F6)
		Patients according to their personality or anxiety have more or less need to be in a paternalistic attitude and others to have information and decide by themselves (. . .) Some patients feel abandoned, and others are more autonomous and capable to decide (F13)

**Table 3.** Individual-centred aspects related to role ascription.

Theme: individual-centred aspects	Selected quotes
ACP requires adaptation to the individual	I adapt to the person. Some need that I ask questions as they are (in the document) and others, I feel, prefer that they are formulated differently (F2) For me the word is flexibility, adaptability according to the specificity of human being, unique and changing (F5)
Degree of self-determination	There are people whose ideas are sufficiently clear. I try to withdraw as much as possible and just make them realise when there are incoherences (. . .) Others clearly need examples. So in that case, I am more of a guide (F6) Patients according to their personality or anxiety have more or less need to be in a paternalistic attitude and others to have information and decide by themselves (. . .) Some patients feel abandoned, and others are more autonomous and capable to decide (F13)
Communication capacity and desires	Sometimes I ask if they want more information and if they say yes, I give them more (F1) We adapt to the patient. If the patient wants statistics, I give statistics (F5)

A final coding book containing all the codes, themes and subthemes was created by ACS. Final interpretation of themes and subthemes was developed by ACS and validated with MB, RJJ (an ethicist and palliative care physician) and ERT. Throughout the process, only information relevant to the objective of the study was coded and interpreted. Coding, theme and subtheme development and interpretation was done in French. The themes, sub-themes and quotations used in this paper were translated from French to English by ACS. We adhered to the Consolidated Criteria for Reporting Qualitative Research (COREQ) checklist,^30^ that we adapted to the specificities of our methodology,^29^ to ensure comprehensive and transparent reporting.

We follow the structural symbolic interactionism approach, developed at the convergence of two different way of understanding professional roles.^31,32^ Structuralists understand roles as essentially fixed positions embedded in the social structure, while symbolic interactionists consider roles as negotiated understandings between individuals and relying on subjective perceptions, therefore always potentially fluctuating. The structural symbolic interactionism approach recognises that professional roles do tend to become institutionalised and that this imposes structural constraints upon them. However, in analysing professional roles, it also recognises the significance of role meaning and perception which influences how roles are enacted and how identity is created.^31,32^ This led our analysis to closely consider how facilitators translate their understanding of what advance care planning requires (the institutional frame) into a stance and set of actual activities (their roles). We refer to ‘role self-ascription’ to recognise the fact that roles are not (solely) those conveyed by the training but are something that professionals build themselves and develop in consideration to their own practice.

## Results

Of 18 identified participants, 14 agreed to participate. They reflect four different professions, both clinical and non-clinical, participated to the study. Table 1 details their characteristics.

Interviews lasted 49 min (mean).

We identified three themes: (1) role typology; (2) individual-centred and (3) professional-centred aspects related to role ascription. All themes and sub-themes are detailed in Tables 2 to 4.

**Table 4.** HP-centred aspects related to role ascription.

Theme: HP-centred aspects	Sub-themes	Selected quotes
Profession		
➢ Profession defines role		I prefer (to identify) as a nurse. (. . .) I am ((name) and I am a nurse and I do ACP in this context (F3) I don’t give medical information because I’m not a doctor (. . .) I can give statistical values on CPR. I can also talk about the chances of success and risks, but not more. I don’t know to what extent one needs detailed medical information. (F10) I use specifically the decision aids from ACP Swiss but also my knowledge after twenty years in intensive care (F12)
➢ Relevance of clinical vs non-clinical professions		I don’t know how people who aren’t doctors or nurses do it, for example social worker, but I find that there are medical questions all the time (F5) The doctor should do (the second part) because I think it’s interesting for a doctor to hear what is being said to approach what comes next (F5) For some medical questions, even if I am a doctor I can’t answer. We had a case (. . .) My role is that I present everything medical, but in my domain of competence. It’s not often but if it exceeds by competence, I need to refer to someone else (F5)
Being involved in the care plan		Patients are asked how they live and whether they want to continue their treatment or not. For someone external it’s easier to ask the question, what really matters for you. And people can say it in ACP, but if the facilitator is involved in the treatment, as doctor or nurse, its harder because they are offering something that is doing good to the patient (F1) Another problem is having the hat of the doctor or the nurse at the same time, and you do the ACP but your patient is hurting, you need to address the pain (F6)

### Theme 1: Role typology

Participants referred to a variety of roles and often to a combination of roles. We identified two overarching role categories: facilitators and counsellors (Table 2), each defined through particular approaches (sub-types) in terms of how communication takes place with patients. The names of the categories were derived from the sub-type that englobed the others.

‘Facilitators’ bestow a complete focus on individual’s autonomy in decision making; they understand their mission as being to empower people to engage in decision-making by offering neutral and complete information and respecting their decisions (‘it’s a philosophy. A holistic history about how I understand care. How I understand companionship in the life process. I don’t think of myself as omniscient. I **accompany** people on the road they want to take and I use my expertise to help’, F8). Facilitators provide a concrete aid also in providing with documentation and helping them to put their preferences into writing, for example by identifying the medical decisions that associate with their preferences. As ‘translators’, they render terms understandable to individuals and the care choices understandable to healthcare professionals. Facilitators don’t give advice about what medical decision might be most suited and don’t decide on the person’s behalf. Keywords associated to this role category comprise of ‘to facilitate’, ‘to accompany’, ‘to translate’, ‘to help’.

‘Counsellors’ recognise their mission as being more directive than that of the facilitator; they give selective information relative to the medical situations and interventions in a way that is considered as relevant for the person, and inform people of what is medically indicated or not (‘I must help the other person to find the right solution’, F9). The counsellor role was also described as related to constructive questioning of individual’s decisions when these don’t reflect their preferences or when they refer to medically irrelevant acts. Keywords associated to this role category comprise of ‘to counsel’, ‘to guide’, ‘to support’, ‘to coach’, ‘to consult’.

This typology is, however, conceptual, since all but one participant referred to endorsing roles from both categories and being, at times, ‘facilitators’, and at others, ‘counsellors’, according to the situation. For example, one participant said, when describing her role, that ‘There are people who are very lost and in those cases one has to be more of a guide, more clear about what is possible. (. . .) There are people who are very determined. In those cases, one just has to listen’ (F4). This highlighted the dynamic of the situation and the adaptability that advance care planning professionals need to show when endorsing their role to certain factors (that are explored as theme 2 and 3).

### Theme 2: Individual-centred aspects related to role ascription

Professionals referred to the fact that their role fluctuates according to the individual (Table 4).

Generic adaptability to the needs of the person was a pre-requisite mentioned by several participants (‘I adapt to the person. Some need that I ask questions as they are (in the document) and others, I feel, prefer that they are formulated differently, F2). Two other aspects in particular were noted. Firstly, the person’s personal ‘degree of self-determination’, that is, to what extent they understand their health situation, have reflected about medical options, and have already made choices. People who have already positioned themselves and are knowledgeable about medical technology and their health situation don’t require much advice but rather a support to translate and transcribe wishes, while those who are less knowledgeable and undecided or haven’t thought of the subject require more active guidance and counselling (‘There are people whose ideas are sufficiently clear. I try to withdraw as much as possible and just make them realise when there are incoherences (. . .) Others clearly need examples. So in that case, I am more of a guide’, F6). Second, the professional’s role was also dependent on the person’s ‘communication capacity and desires’, that is to what extent they welcomed and requested certain information, as well as their ability to process it (‘We adapt to the patient. If the patient wants statistics, I give statistics’, F5).

### Theme 3: Professional-centred aspects related to role ascription

Professionals also referred to intrinsic aspects that define their role (Table 4).

Participants referred to their ‘main profession’ as bearing weigh on their role. For some, their clinical defined their role during the advance care planning, as they drew on knowledge and attitude particular to that profession (‘I prefer (to identify) as a nurse. (. . .) I am ((name)) and I am a nurse and I do ACP in this context’, F3). One participant found that not being a clinician might impact the role in advance care planning, for example about what information they can (or cannot) give to the person (‘I don’t know how people who aren’t doctors or nurses do it, for example social worker, but I find that there are medical questions all the time’, F5). Certain professions were considered more competent to conduct advance care planning, such as being a clinician or a medical specialist. Another aspect was related to ‘being involved in the care plan’ of the person. Here, participants noted the challenges of ‘double hatting’ (being the patient’s treating doctor and doing an advance care planning with them): individuals might be less adamant to refuse treatments that facilitators suggest, and facilitators need to prioritise symptom management, which might be disruptive for advance care planning.

## Discussion

### Main findings

This study provides evidence about how professionals self-ascribe their role within advance care planning discussions by exploring accounts of how they perceive, define and describe it. Participants provided rich accounts of their stance, which confirms that professionals feel the need to better define their roles within the process.^6,19,20^

### What this study adds?

Our findings highlight that roles fluctuate according to various dimensions but can be aggregated in two overarching categories, ‘facilitators’ and ‘counsellors’. ‘Facilitators’ consider people as experts and knowledgeable about what is relevant or not in terms of medical risk-taking, whereas ‘counsellors’ undertake a more custodial role, and design information and explanations in a way that explicitly describes what is medically relevant. While facilitators put the onus on autonomous decision-making (prioritising the person’s right to document their choice independent of medical indications), counsellors focus more on informed decision-making (prioritising the person’s need to receive information that is personalised and a clear explanation of what is medically indicated).

This dual polarisation is by far not representative for most participants, who rather referred to their role shifting dynamically between these two categories, showing that role borders are permeable and that they juggle with a dual attitude. Role ascription is anchored in what the individuals themselves make relevant as needing, as well as in the identity and position of the professional themselves. Professions were deemed important to the point to which some participants didn’t initially acknowledge endorsing another advance care planning -specific role than that of their clinical professions. Professionals who do advance care planning with their patients experience certain challenges in maintaining their role (and continuing advance care planning) when patients had symptoms in need of tending. All this shows that competences and stances from main professions overflow the role of professionals. This begs the question of to what extent certain desirable competences for advance care planning professionals are found within specific professions, and, more globally, of the place of communication as professional competence.^33^ For example, prior evidence shows that some of the barriers encountered by professionals concern the fear of depriving patients of hope by way of presenting options,^34^ which is a core skill for initiating end-of-life discussions. While a call has been made for advance care planning research to focus more on the ‘diversities and variations between people’, it is implicit that there is equally a need to address how facilitators face diversity and adapt to it.^35^

Role fluctuation can be challenging, since it requires a lot of adaptation and flexibility. Role boundaries are permeable, with specific practices or activities being conjugated in a way that tend more towards one role than another. It also means that professionals don’t have a unique professional identity associated to advance care planning, which might make their experience more complex.

### Implications for practice

Thinking about role specificity is the only way to identify appropriate resources for professionals. Training can directly address the issue of role ascription and the link between original profession and advance care planning role. Ibarra and Obodaru^36^ highlight, to this regard, the importance of the concept of ‘liminality’, that is, the experience of role transition, or rather when a person feel ‘betwixt and between’ roles. Ibarra and Obodaru argue that liminal experiences can be more or less institutionalised, and, in particular, that under-institutional liminal experiences are subjectively more challenging for individuals (because they have less guidance and support) but have a greater potential for identity growth (since they allow more room for individual agency and creativity). More research is necessary to explore dimensions such as shifting from professional roles to ACP roles as well as shifting between the typologised ACP roles.

### Strengths and limitations

This study explores how professionals self-ascribe roles during advance care planning discussions, a topic that has been under-researched. Its strengths reside in the diversity of participants (clinicians and one non-clinician), and in the inductive approach used for data analysis, that allowed to be faithful to participants’ experience. This study also has limitations. Firstly, a greater number of participants would have allowed to reach a more granular analysis. Indeed, role flexibility generates a diversity of patterns in which participants would experience and understand their roles. Furthermore, we only captured participants’ subjective point of view, but we acknowledge that a more naturalistic approach would be more informative (e.g. relying on recorded advance care planning conversations) to develop an understanding of role dynamics. Only one of the participants was not a clinician. Since profession seems to be an important factor in role ascription, important questions related to the relevance of the clinical background are eschewed. Finally, the sample size of healthcare professions other than nursing (4/14) is too small to generalise with confidence. However, given that ACP is overwhelmingly done by nurses in the jurisdiction from which the participants were drawn, this somewhat mitigates the concern.

## Conclusions

Roles that professionals undertake during advance care planning encounters can be aggregated in two overarching categories, ‘facilitators’ and ‘counsellors’, according to whether they prioritise autonomous decision-making or informed decision-making. In practice, however, roles fluctuate between these two categories, according to individual-related aspects (to what extent the person is informed and eager to engage in autonomous decisions, and what their communication capacity and desires are) or professional-related aspects (their clinical or non-clinical profession and their involvement in the individual’s care plan). This means that advance care planning requires professionals to be very adaptable and flexible throughout the process in order to identify the role that they can invest in each situation. Additionally, our results also show that the professionals themselves distinguish between the roles of facilitator and counsellor, which has ethical implications in terms of responsibility. Training and further research need to take into consideration this complexity and address it explicitly.

## Supplemental Material

sj-docx-1-pmj-10.1177_02692163251331168 – Supplemental material for Role self-ascription of professionals conducting advance care planning conversations: A thematic analysis Supplemental material, sj-docx-1-pmj-10.1177_02692163251331168 for Role self-ascription of professionals conducting advance care planning conversations: A thematic analysis by Anca-Cristina Sterie, Mathieu Bernard, Ralf J Jox and Eve Rubli Truchard in Palliative Medicine
