# Supplementary material for: Role self-ascription of professionals conducting advance care planning conversations: A thematic analysis
Source: Palliat Med. 2025 Apr 25;39(6):700–8. doi: 10.1177/02692163251331168 (PMC12102511; doi:10.1177/02692163251331168)
Supplement: sj-docx-1-pmj-10.1177_02692163251331168 – Supplemental material for Role self-ascription of professionals conducting advance care planning conversations: A thematic analysis [file sj-docx-1-pmj-10.1177_02692163251331168.docx]

Supplementary Material

1. Interview guide

Part 1. Socio-demographic and professional information: nationality, professions, place of work, current position

Part 2. ACP training and practice

- What kind of training have you had related to ACP?
- What other kind of training related to communication about medical decisions and end of life do you have?
- In what professional context have you started to work with ACP?
- What inspired you to integrate ACP in your practice?
- How have you been involved in ACP, since your training was over?
- For how long have you been doing ACP?
- In what languages do you do ACP?
- How many beneficiaries have you accompanied for ACP?
- What kind of beneficiaries do you do ACP with (in terms of setting, diagnosis, age…)?
- Please describe how you usually conduct an ACP, in terms of how you contact beneficiaries (or are contacted by them), where you conduct discussions, who participates, how long it usually lasts (one session and in number of sessions), whether you follow a specific structure and/or document
- Do discussions always have the same structure? What dictates their structure?
- Do you use a guide for the discussion? Which? Have you adapted it (and how)?

Part 3. Perceptions about ACP

- What does ACP mean for you?
- How do you understand your role within ACP? Has something changed in how you understand your role, comparing to the beginning of your practice?
- In particular, how do you understand your role within ACP related to the part about medical decisions?
- How do you understand your role within the relationship between beneficiary and relative?

Part 4. Communication

- How do you usually explain the part about medical decisions to beneficiaries? Can you give me an example?
- What kind of information do you give or not give to beneficiaries, related to medical decisions? How do you decide what kind of information to give? How did you come up with that information?
- Do you have any conversational resources for doing ACP that you’ve developed with time? Which ones? How do you employ them?
- Is there something with which you’re not at ease and talking with beneficiaries? How has this evolved with time and experience?
- Is there something that is / used to be a challenge for you when doing ACP and talking with beneficiaries? How do you / did you tackle these challenges? What changed?
- Are there situations in which you didn’t agree with the decision that the beneficiary made? How do you deal with these situations?
